# Editorial: Neuromorphic and deep learning paradigms for neural data interpretation and computational neuroscience

**DOI:** 10.3389/fncom.2026.1789388

**Published:** 2026-02-13

**Authors:** Chenglong Zou, Rui Yuan, Jun Wen

**Affiliations:** 1 School of Integrated Circuits, Beijing University of Posts and Telecommunications, Beijing, China; 2 College of Computer Science, Chongqing University, Chongqing, China; 3 Department of Neurosurgery, Xinqiao Hospital, Army Medical University, Chongqing, China; 4 College of Artificial Intelligence, Southwest University, Chongqing, China; 5 Department of Computational Biology, Mohamed bin Zayed University of Artificial Intelligence, Abu Dhabi, United Arab Emirates

**Keywords:** brain-inspired computing, deep learning, neural data interpretation, neuromorphic computing, spiking neural networks (SNNs)

Interdisciplinary research between neuromorphic and deep learning paradigms has developed rapidly in recent years. Key advances include silicon nano-devices and memristors for neuromorphic computing, brain-computer interfaces (BCIs), and high-performance SNN models for neural decoding. These technologies have significantly advanced the application of neuroscience discoveries in medicine and engineering. Based on this observation, we initiated a Research Topic and received a total of seven submissions. Finally, four distinguished articles were accepted for publication after rigorous peer review. We would like to express our sincere thanks and congratulations to all the authors and reviewers who collaborated on this Research Topic. The following contributions and highlights will be relevant to researchers in this field.

## Contribution 1: DT-SCNN: dual-threshold spiking convolutional neural network with fewer operations and memory access for edge applications

The article by Lei et al. introduces a novel Dual-Threshold Spiking CNN (DT-SCNN) designed for edge applications. It uses a dual-threshold LIF neuron to generate two spiking feature maps from one membrane potential map, thereby halving the necessary operations, weights, and memory access. The results show a reduction of approximately 50% in convolutional operations with minimal accuracy loss (< 0.4%) on CIFAR10/MNIST/Fashion-MNIST, enabling efficient and low-latency edge deployment.

## Contribution 2: Neuromorphic energy economics: toward biologically inspired and sustainable power market design

Ye et al. propose a neuromorphic computing-inspired paradigm for sustainable power market design. The study advocates using event-driven SNNs for microsecond-scale, energy-efficient dynamic pricing and grid management. The authors suggest that enabling decentralized and self-organizing coordination of distributed energy resources could enhance grid resilience to renewable energy volatility.

## Contribution 3: Triboelectric nanogenerators for neural data interpretation: bridging multi-sensing interfaces with neuromorphic and deep learning paradigms

Gan et al. review the Triboelectric Nanogenerators (TENGs) as self-powered, flexible multi-sensors for acquiring neural and physiological signals (e.g., EEG and EMG). The authors highlight the ability of deep learning models (e.g., CNNs and RNNs) to converge for data interpretation and the advantages of neuromorphic computing methods for ultra-low-power, event-driven processing. Finally, this synergy is positioned as crucial for advanced elderly health monitoring and brain-computer interfaces.

## Contribution 4: Bridging neuromorphic computing and deep learning for next-generation neural data interpretation

The study by Zhang et al. proposes a hybrid framework that integrates neuromorphic computing and deep learning for neural data interpretation. The authors argue that it is necessary to combine an event-driven, low-power neuromorphic front end (e.g., SNNs) for spike-based processing with a powerful deep learning back end for high-level pattern recognition. This work also emphasizes the importance of balancing biological plausibility, energy efficiency, and computational performance.

Overall, the contributions presented in these studies focus on the integration and synergy of deep learning and neuromorphic computing. Lei et al. presents a high-accuracy and low-cost SCNN for edge applications. Ye et al. demonstrates the potential of neuromorphic methods for self-organizing, decentralized, and robust energy management. Gan et al. calls for the integration of the advantages of deep learning and neuromorphic computing for future health monitoring and brain-computer interfaces. Finally, Zhang et al. recommends bridging neuromorphic computing and deep learning for next-generation neural data interpretation, such as pattern recognition. Each contribution provides significant advancements in its respective field, demonstrating the potential of neuromorphic computing and deep learning techniques in practical applications.

